# One-Step Microwave-Assisted Fabrication of Carbon Dots as Efficient Fluorescent Chemosensors for Hg^2+^ and Fe^3+^ Detection

**DOI:** 10.3390/s25247452

**Published:** 2025-12-07

**Authors:** Rawan H. Alansari, Esraa M. Bakhsh, Lenah R. Altamimi, Kalsoom Akhtar, Sher Bahadar Khan

**Affiliations:** Chemistry Department, Faculty of Science, King Abdulaziz University, P.O. Box 80203, Jeddah 21589, Saudi Arabia; ralansari0044@stu.kau.edu.sa (R.H.A.); laltamimi0005@stu.kau.edu.sa (L.R.A.); kshergul@kau.edu.sa (K.A.); sbhkhan@kau.edu.sa (S.B.K.)

**Keywords:** carbon dots, chemosensor, Hg^2+^, Fe^3+^, detection

## Abstract

Fluorescent carbon dots (CDs) were efficiently synthesized by a one-step microwave-assisted method using diphenylamine as a carbon precursor. The obtained CDs exhibit high stability and strong water solubility. Under UV irradiation, these CDs could emit bright green photoluminescence. These synthesized CDs have an average diameter of 1.8 nm (±0.46) and quantum yield (QY) as high as 44.69% using rhodamine-B as a reference. The CDs’ intensity can be quantitatively quenched by Hg^2+^ and Fe^3+^ ions with high sensitivity and low LOD about 9.58 nM and 22.27 nM, respectively, indicating that the CDs sensors can be potentially applied for Hg^2+^ and Fe^3+^ detection in aqueous solutions.

## 1. Introduction

As a category of zero-dimensional carbon-based nanomaterials, carbon dots (CDs) have been widely applied in chemosensing, photocatalysis, bioimaging, and many applications due to their excellent stability, biocompatibility, and optoelectronic properties [1]. CDs are generally known to be discrete, quasi-spherical particles with sizes below 10 nm, have a conjugated core (sp^2^) superposed by functional groups such as aldehyde, carboxyl, ketone, etc., which depends on the nature of the carbon resource material used to prepare the CDs [2]. CDs possess unique optical properties such as excellent photoluminescence, tunable fluorescence, as well as low toxicity, chemical inertness, good biocompatibility, and the ability to undergo photoinduced electron transfer [3,4,5]. CDs are a new category of zero-dimensional fluorescent nanomaterials that consist of conjugated carbon nuclei and surface functional groups mainly composed of elements C, H, and O doped with heteroatoms B, N, S, F, P, and Cl [6,7,8]. CDs can be synthesized through a number of approaches, which mainly include top-down and bottom-up methods [4,9]. The top-down technique is breaking down the larger carbon materials to the nanoscale using methods like electrochemical oxidation, ultrasonic synthesis, laser ablation, and arc discharge [10,11,12,13,14,15]. Generally, these methods require long processing times, harsh conditions, and expensive equipment. Conversely, bottom-up methods known as building CDs from starting material such as biowastes, phenyldiamine, citric acid, or glucose using techniques like hydrothermal carbonization, chemical oxidation, combustion, pyrolysis, and microwave-assisted synthesis [10,16,17,18,19,20,21,22,23,24]. Bottom-up synthesis offers better control of size, composition, and fewer imperfections compared to top-down methods [10]. Among these techniques, the microwave-assisted method has emerged as a highly efficient and eco-friendly technique for CDs synthesis [25,26,27,28,29]. This approach involves rapid heating of carbon-rich precursors such as biowaste using microwave irradiation, which enhances carbonization and improves product yield [30]. The reaction parameters, such as irradiation time, microwave power, and precursor concentration, influence the optical and morphological properties of the produced CDs [31]. CDs have become outstanding materials in the development of fluorescent chemosensors for heavy metal ion detection due to their unique photoluminescent properties, high surface area, and tunable surface functionalization [32,33,34,35]. The heavy metal ions interact with the surface groups of CDs, leading to fluorescence quenching or enhancement changes [36].

On the other hand, heavy metal ions are essential for maintaining human health because many biological functions depend on the presence of these ions, and many enzymes require these elements for their catalytic reactions [29]. Mercury is a neurotoxin impacting both humans and animals, particularly Hg^2+,^ which is the most stable form of mercury [37]. It is commonly recognized that the excessive release of mercury ions not only endangers the ecological environment but also leads to serious damage to human health through direct absorption and bioaccumulation through food chains. People are likely to be exposed to higher mercury doses under specific and non-specific conditions when using mercury-containing ingredients, such as traditional medicine or cosmetic skin products. Accidental exposure can be possible when air and water pollution, and home mercury spills occur [37].

Iron is an essential element in the environment and exists mainly in two oxidation states: Ferrous (Fe^2+^) and Ferric (Fe^3+^). While iron is important for biological processes such as oxygen transport and enzymatic reactions, its presence in water with high concentrations can pose significant hazards. Excess iron can lead to contamination of drinking water. More significantly, high iron concentrations may raise the growth of iron bacteria, which can block the water systems and contribute to the formation of biofilms, potentially harboring pathogenic microorganisms [38]. High levels of iron in water can cause immediate harmful effects on health, such as organ damage and gastrointestinal irritation. However, it can interact with other contaminants like arsenic, making it easier for them to be released and raising the risk of contamination [39].

Pollutant detection in aquatic environments is one of the most significant applications of CDs, and researchers are currently conducting many studies on this unique material to develop its properties in order to use it in many fields. This study aims to develop a fluorescent chemosensor using a simple and effective microwave-assisted method and use it to detect Hg^2+^ and Fe^3+^ ions in aquatic environments with low LOD.

## 2. Materials and Methods

### 2.1. Chemicals

Orthophosphoric acid, mercury (II) nitrate hydrate, sodium thiosulphate pentahydrate, silver nitrate, strontium chloride, lead nitrate, cadmium nitrate, and iron (II) sulfate heptahydrate were purchased from BDH Chemicals Ltd. (Poole, UK). Diphenylamine, zinc sulfate heptahydrate, lanthanum (III) nitrate hydrate, sodium chromate, magnesium chloride, and sodium hydroxide were purchased from Sigma Aldrich (Darmstadt, Germany). Copper (II) sulfate pentahydrate was purchased from Philip Harris, nickel sulfate from Lobachemie, and ferric chloride anhydrous from SRL (Mumbai, India). Hydrochloric acid was purchased from Honeywell (Seelze, Germany). All chemicals used are of analytical grade and as received without any further purification. Deionized water was used in all experiments prepared in the laboratory using the (PURELAB) instrument (Bolton, UK).

### 2.2. Instrumentation

A microwave oven from Nikai (20 Liter 700 W with Auto Menu Function| Model No. NMO515N8NX) (Jinhua, China) was used. Fourier transform infrared (FTIR) spectra were recorded at room temperature using an FTIR spectrometer from Perkin Elmer (Shelton, CT, USA), and the UV-vis absorption spectra were obtained using a spectrophotometer from Thermo Scientific (Shelton, CT, USA). The PL emission spectrum was measured using a Cary Eclipse Fluorescence Spectrometer from Agilent (Santa Clara, CA, USA). A pH meter from JENWAY (Dunmow, UK) was used for pH measurements. TEM-EDS with a specimen quick-change holder (EM-11210SOCH) from JEOL Ltd. (Akishima, Tokyo, Japan) was used to provide morphological and elemental analysis.

### 2.3. Synthesis of CDs

**Scheme 1:**
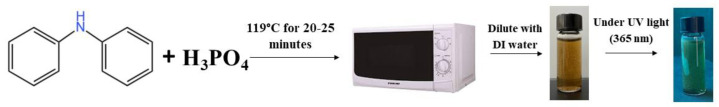
Schematic representation for preparation of CDs.

An amount of 0.50 g of diphenylamine was added to 10 mL of phosphoric acid and dissolved using stirring and heating until completely dissolved. They were mixed and then placed in a microwave-safe container and heated in a microwave oven at ~119 °C for 20–25 min (until the solution turns dark). The dark solution was then dialyzed to produce CDs (Scheme 1). CDs were diluted to 500.00 mg L^−1^ for use as a sensor, since it can retain sufficient fluorescence intensity for sensing. Lower concentrations (such as 250.00 mg L^−1^) may reduce signal intensity, while higher concentrations (such as 1000.00 mg L^−1^) could lead to aggregation or self-quenching [40].

### 2.4. Detection of Hg^2+^ and Fe^3+^

**Scheme 2:**
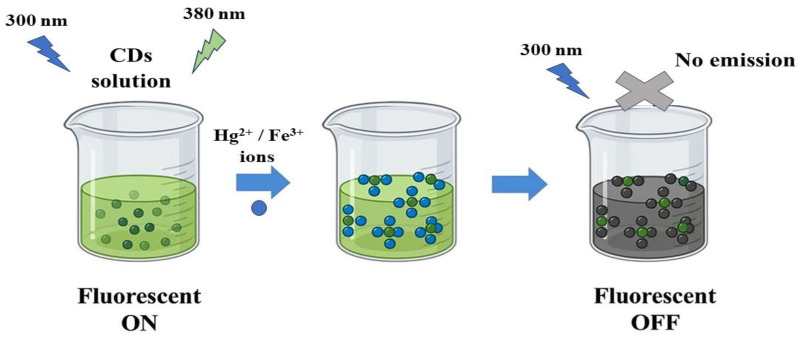
Schematic representation for the quenching of CDs intensity by Hg^2+^ and Fe^3+^ ions.

The selectivity of Hg^2+^ and Fe^3+^ ions was performed by adding other metal-ion solutions: (Na^+^, Ag^+^, Zn^2+^, Cr^6+^, Fe^3+^, Fe^2+^, Cd^2+^, La^3+^, Pb^2+^, Cu^2+^, Ni^2+^, Sr^2+^ and Mg^2+^) in a similar way. Stock solutions (50.00 μM) were prepared to study the selectivity of the CDs towards the heavy metal ions. Then, standard solutions with different concentrations (5.00, 10.00, 50.00, 100.00, 150.00, 200.00, 400.00, 600.00, 800.00, and 1000.00 μM) of the selected heavy metal ions, which showed quenching effect, were prepared to study their behavior and interactions with the CDs. All experiments were performed at room temperature. Scheme 2 illustrates the quenching process by Hg^2+^ and Fe^3+^ ions. 

### 2.5. Measurement of Fluorescence Quantum Yield (QY)

The QY of CDs was calculated using the following equation:

$$\Phi =\Phi_{R}\times \frac{I}{I_{R}}\times \frac{A_{R}}{A}\times \frac{\eta^{2}}{\eta_{R}^{2}}$$

where

Φ = Quantum yield, *I* = Intensity, η = Refractive index, A = Absorption, and R subscript represents the fluorescence reference of known quantum results. Rhodamine-B (aqueous solution, Φ_R_ = 0.31) was used as a reference. The CDs were dissolved in deionized water (η = 1.33) as preparation for the measurements.

### 2.6. Limit of Detection (LOD) and Limit of Quantification (LOC) Estimation

LOD = 3.3 σ/s and LOC = 10 σ/s, where σ and s are related to the blank sample’s standard deviation and slope of the S-V plot, respectively.

### 2.7. Relative Fluorescence Change (F − F_0_)/F_0_

The fluorescence response of the prepared CDs to heavy metal ions was quantitatively assessed by measuring the relative change in fluorescence intensity, which is defined as (F − F_0_)/F_0_, where F_0_ represents the initial fluorescence intensity in the absence of target heavy metal ions, and F is the fluorescence intensity in the presence of heavy metal ions. This relative fluorescence change is a key metric for evaluating the selectivity of sensors towards heavy metal ions, as significantly different (F − F_0_)/F_0_ values for various metal ions indicate a sensor’s ability for a specific metal detection. A sensor with high selectivity will show a large (F − F_0_)/F_0_ change for its target metal and a small change for other non-target ions. This change can have positive or negative values:

- Fluorescence Enhancement: In some cases, the metal ion can cause an “on–off” fluorescence response, where the fluorescence increases. In this case, F > F_0_, and (F − F_0_)/F_0_ becomes a positive value.
- Fluorescence Quenching: When a metal ion interacts with a sensor, it can quench the sensor’s fluorescence intensity. This leads to a decrease in F_0_ value, making (F − F_0_)/F_0_ a negative value. (Common in heavy metal ion detection).

### 2.8. F/F_0_

The F/F_0_ ratio provides a direct measurement of fluorescence intensity changes. It is commonly used in fluorescent sensor studies to quantify changes in the intensity relative to the initial intensity.

### 2.9. Relative Fluorescence Intensity (F_0_ − F)/F_0_

A relative fluorescence intensity plot quantifies the change in fluorescence signal relative to the initial intensity, often used to measure the impact of an analyte on a fluorescent probe. This plot indicates the fractional decrease in fluorescence and is commonly used in sensor development to determine analyte concentrations or changes in environmental conditions.

### 2.10. Stern–Volmer Plot

The Stern–Volmer equation is:

F_0_/F = 1 + K_SV_ [Q]

where [Q] is the quencher concentration and K_SV_ is the Stern–Volmer quenching constant, representing the efficiency of quenching. This ratio allows the evaluation of quenching efficiency, elucidation of quenching mechanisms (static or dynamic), and quantification of analyte–quencher interactions, which will provide key parameters for sensor performance.

### 2.11. Benesi–Hildbrand Plot

The Benesi–Hildebrand (B-H) plot is a graphical method used to determine the stoichiometry and equilibrium constant (binding constant) of host–guest complexes, or other non-bonded interactions, by analyzing changes in fluorescence intensity. *Y*-axis (1/(F_0_ − F)) represents the inverse of the change in the observed fluorescence intensity upon complex formation. *X*-axis (1/[Q]) represents the inverse of the concentration of the guest molecule (heavy metal ions), which is added to the host (CDs). The linear plot confirms the assumption of a 1:1 stoichiometry and allows for the calculation of the association constant (K), which represents the strength of the interaction between the molecules from the intercept and slope. A nonlinear plot indicates that the interaction is not a simple 1:1 complex and suggests the formation of a 1:2 or higher-order complex.

### 2.12. Study of CD Stability

#### 2.12.1. pH Stability

HCl and NaOH were used to prepare CD solutions with different pH values ranging from 2 to 13. Fluorescence intensities were measured after 15 min.

#### 2.12.2. Thermal Stability

To study the thermal stability, the temperature of the CD solutions was adjusted between 20 and 100 °C. The fluorescence intensity was measured immediately after the CD solutions reached the desired temperature.

#### 2.12.3. Storage Time Stability

CD solutions were stored in tightly closed containers at room temperature to prevent contamination, aggregation, or changes in properties.

## 3. Results and Discussion

### 3.1. Characterization of CDs

**Figure 1:**
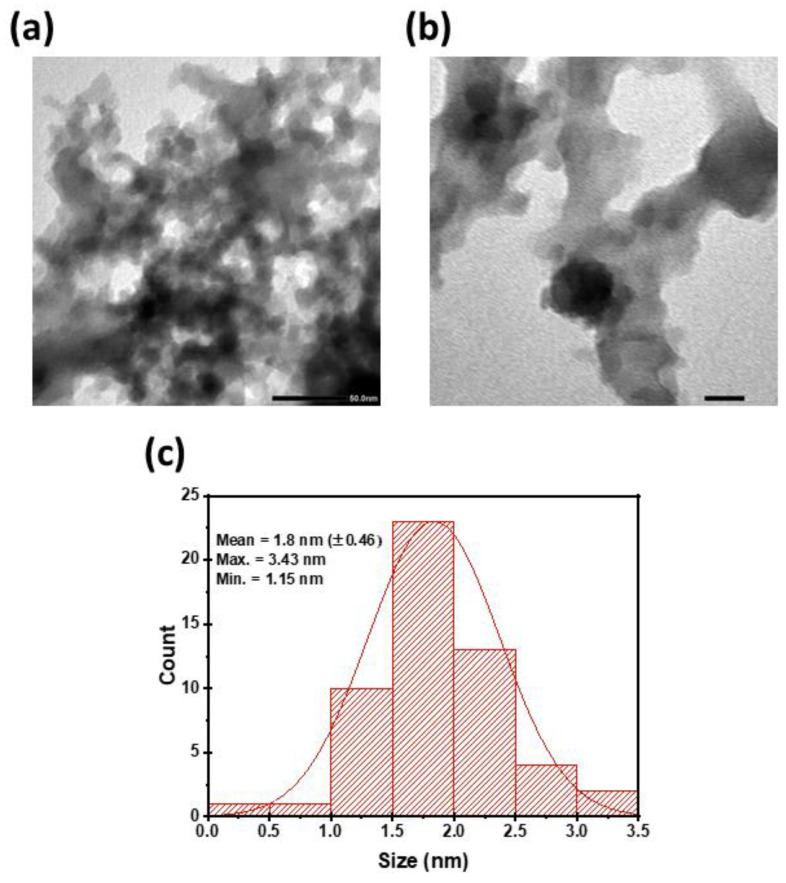
TEM image of CDs at (**a**) 50.00 nm, (**b**) 20.00 nm; (**c**) particle size distribution.

To evaluate the particle size distribution of the prepared CDs, TEM analysis was applied. Figure 1a,b showed that the CDs have an average diameter of 1.80 (±0.46 nm). The size range extended from 1.15 nm to 3.43 nm as demonstrated in Figure 1c, which indicates a relatively narrow distribution and good uniformity. These findings suggest the synthetic approach produces CDs with consistent dimensions, which is helpful for applications that require nanomaterials with a uniform size. 

**Figure 2:**
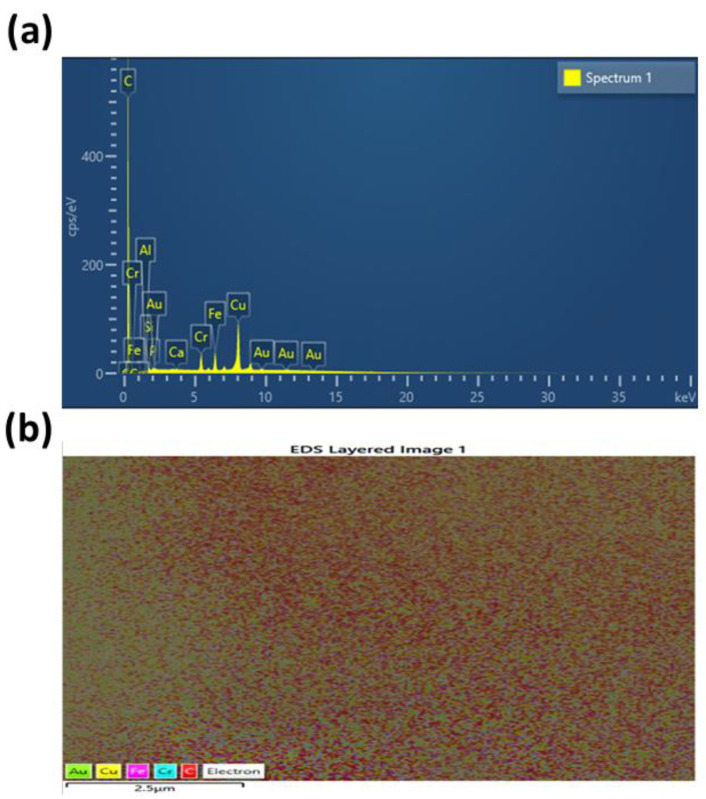
(**a**) EDS and (**b**) mapping analysis of the prepared CDs.

The EDS technique has been applied to obtain the elemental composition of the synthesized CDs, as shown in Figure 2a. The obtained findings illustrated that carbon was the dominant component (70.65 wt%) with a minor phosphorus contribution (0.29 wt%) corresponding to phosphorus doping using o-phosphoric acid. Other elements have values of: Al 0.20%, Si 0.42%, Ca 0.21%, Cr 4.32%, Fe 5.43%, Cu 16.50%, and Au 1.98%. The Au signal results from gold coating applied prior to EDS analysis, while metallic element residues reflect environmental and instrumental influences rather than compositional features of the precursors. Generally, the EDS analysis confirms successful preparation of carbon-rich, phosphorus-functionalized CDs. 

The EDS layered mapping analysis in Figure 2b demonstrates the spatial distribution of elemental species within the carbon dot sample. Each color represents a specific element as indicated in the legend. The dominant red coloration observed across the mapped area indicates a homogeneous distribution of carbon, which is consistent with the formation of carbon dots. Minor signals from Au, Cu, Fe, and Cr, marked by their respective colors, result from either substrate background, sample holder, or possible trace residues from reagents and synthesis vessels. This mapping confirms the successful synthesis of CDs with a uniform elemental composition. The presence of carbon as the continuous phase affirms that the CDs are well dispersed without significant aggregation or phase separation. The spatial homogeneity is further corroborated by the lack of distinct clusters, ensuring consistent fluorescence behavior throughout the sample. Although nitrogen appears in FTIR analysis, it often does not appear in EDS analysis due to its low atomic number, which results in weak X-ray emissions that are easily absorbed by the detector window or surface contamination. In addition, nitrogen must be present above a certain concentration (around 2.00% by mass) to be detected, and this makes its EDS detection unreliable for low levels in many samples [41].

**Figure 3:**
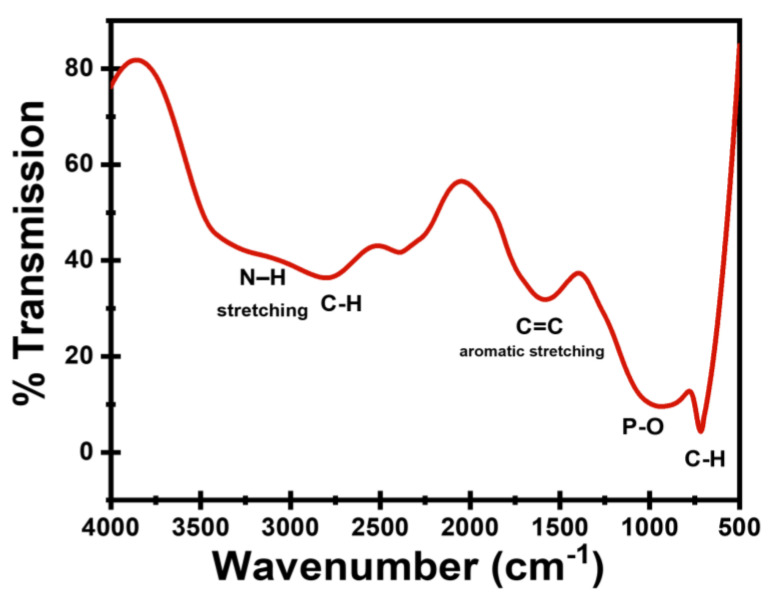
FTIR spectrum of the prepared CDs.

FTIR spectrum of the as-prepared CDs in Figure 3 showed a medium prominent band at 715 cm^−1^ contributed to aromatic C–H out-of-plane bending or P–O bending modes. There is a fairly strong peak at ~1000 cm^−1^ attributed to P–O/P=O/P–O–C vibrations and C–O (ether/ester) stretches, P–O vibrations given owing to the use of o-phosphoric acid. A high moderate peak at ~1600 cm^−1^ is assigned to C=C aromatic stretching. A medium to moderate peak at 2810 cm^−1^ is attributed to aliphatic C–H stretching. Finally broad and shallow peak at 3000–3400 cm^−1^ suggests hydroxyls (surface OH) and possible amine N–H stretching (from Diphenylamine).

**Figure 4:**
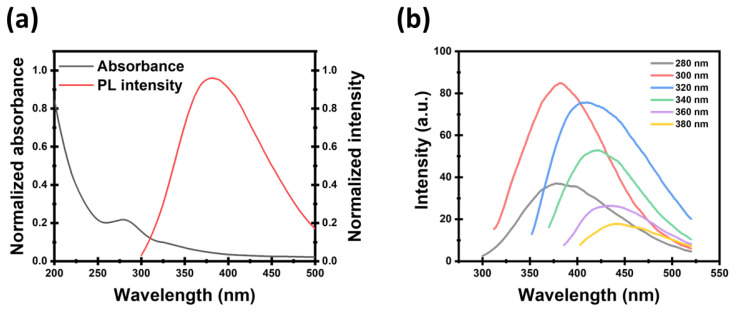
(**a**) Absorption and PL intensity spectrum of CDs. (**b**) Emission PL spectrum at different exciting wavelengths.

The optical properties of the prepared CDs were characterized by UV-Vis and PL intensity, as shown in Figure 4a. The absorbance spectrum exhibits a distinct peak at 280 nm, indicating the characteristic electronic transitions in the CDs. The corresponding PL intensity spectrum, recorded with excitation at 280 nm, shows an emission peak centered around 380 nm, confirming the strong photoluminescent property of the CDs under UV excitation [42]. Also, it displays the PL intensity peak of CDs. Excitation-dependent emission has been illustrated by the prepared CDs, suggesting that the fluorescent/luminescent wavelength can be changed by varying the excitation wavelength. Figure 4b shows the emission PL spectra of CDs, with different excitation wavelengths within the range from 280 to 380 nm.

The QY of the prepared CDs was calculated to be 44.69%. This value is significantly higher compared to CDs in the previous studies (Table 1), which proves the high efficiency of the microwave-assisted technique. This high value suggests that the adopted approach enables effective surface passivation of the CDs, which makes them suitable for different applications in sensing and bioimaging.

### 3.2. Stability of CDs

**Figure 5:**
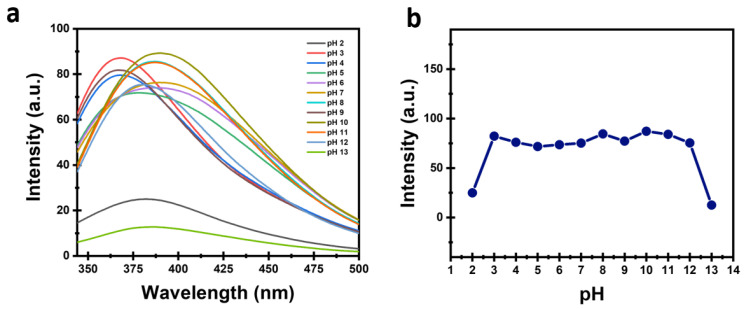
(**a**,**b**) PL intensity stability of CDs at different values of pH.

The stability of CDs is one of the significant parameters for employing them in sensing applications. Thus, the pH stability of the CDs was studied over a broad pH range (pH 2–13) as shown in Figure 5. The PL intensity of the prepared CDs exhibited remarkable stability in a wide pH range (4–11), while a notable decrease in PL intensity was observed under strongly acidic (pH 2) and basic (pH 12–13) conditions. This behavior can be attributed to the protonation and deprotonation of surface functional groups such as COOH, OH, and NH_2_. At lower pH, protonation of these groups introduces non-radiative recombination pathways, which result in PL intensity quenching. Conversely, in highly basic conditions, deprotonation changes the surface charge and may promote partial aggregation, also reducing the PL intensity. The slight shift in emission wavelength observed with pH variation arises from modifications in the surface state energy levels, which affect the electronic transitions responsible for emission. Generally, the CDs demonstrate excellent PL stability over a broad pH range, indicating robust surface passivation and stable optical properties, and significant potential of the CDs in several applications.

**Figure 6:**
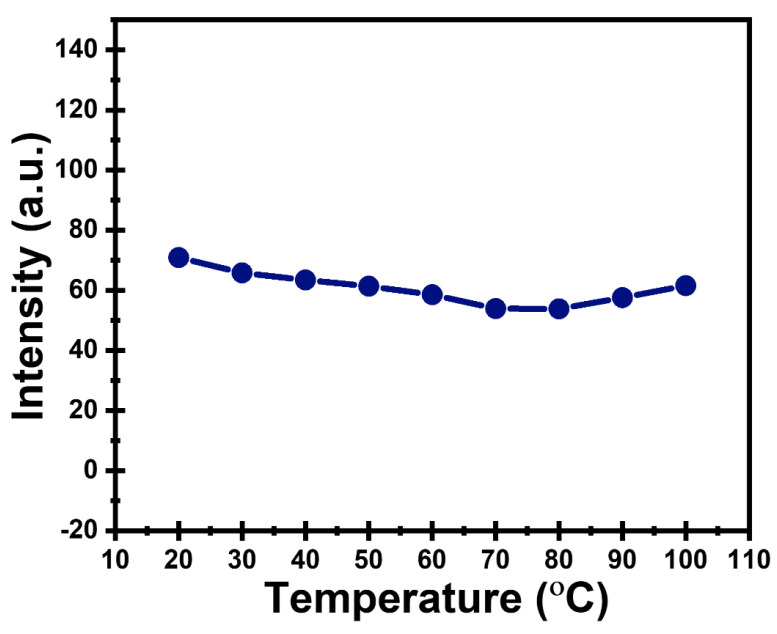
Temperature-dependent PL intensity of CDs.

The PL intensity of the prepared CDs gradually decreased with increasing temperature (Figure 6), indicating typical thermal quenching behavior. The PL intensity of the prepared CDs gradually decreased with increasing temperature, indicating typical thermal quenching behavior. This decrease is attributed to enhanced non-radiative relaxation processes such as phonon-assisted recombination, which become more pronounced at higher temperatures and compete with radiative emission. In addition, elevated temperatures may slightly disturb the surface passivation of the CDs, allowing charge carriers to escape from emissive surface states, thus reducing fluorescence efficiency. Generally, the CDs exhibit good thermal stability, maintaining consistent emission characteristics within a broad temperature range [51].

**Figure 7:**
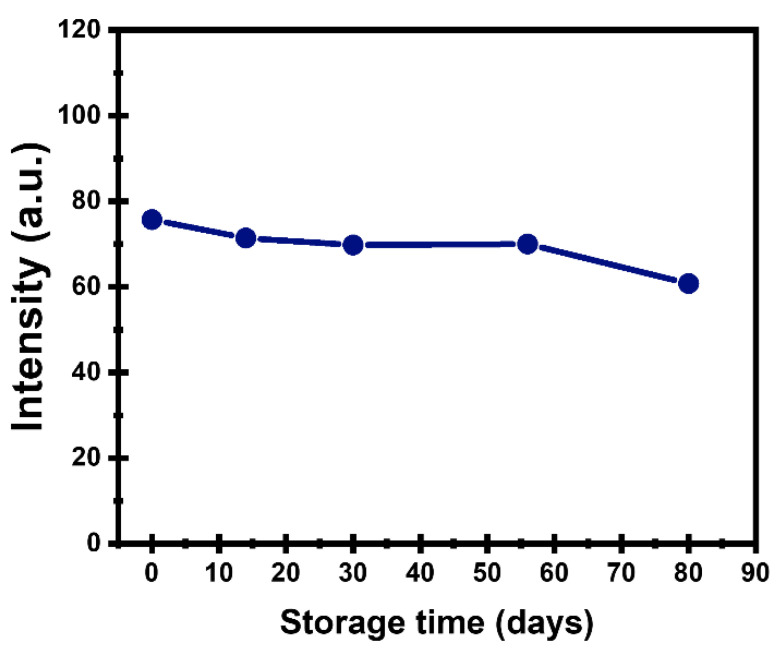
PL intensity of CDs stored at room temperature.

According to Figure 7, the PL intensity of the prepared CDs retained approximately 80% of its original value after 80 days of storage, indicating good storage stability under the studied conditions.

### 3.3. Selectivity of CDs Toward Hg^2+^ and Fe^3+^

**Figure 8:**
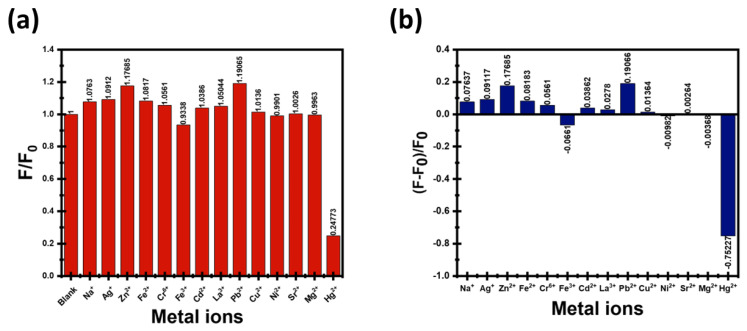
(**a**) Histogram of CDs intensity with several metal ions; (**b**) Fluorescence response of CDs in the presence of different metal ions.

As illustrated in Figure 8a, the CDs’ fluorescence intensity could be significantly quenched by Hg^2+^ and Fe^3+^ compared with the other metal ions. Figure 8b illustrates the fluorescence response of CDs in the presence of different metal ions at $\lambda_{max}$ = 380 nm. The ratio (F − F_0_)/F_0_ is commonly used to quantify the relative change in the CDs’ fluorescence intensity when metal ions are introduced. This value represents the fractional change in fluorescence and is particularly helpful for comparing the sensitivity and selectivity of CDs with different metal ions. A negative value indicates fluorescence quenching (decrease in intensity), like Hg^2+^ and Fe^3+^ ions. The positive value indicates fluorescence enhancement (increase in intensity). Here, the (F − F_0_)/F_0_ was calculated for each metal ion. As shown, Hg^2+^ induced the most significant quenching, with a value of −0.752, and Fe^3+^ with a value of −0.066. While other ions exhibited no quenching effect.

Diphenylamine-derived CDs contain nitrogen-rich surface functionalities from the precursor itself, which facilitate strong ligand-to-metal interactions with Hg^2+^ and Fe^3+^. The presence of nitrogen or other heteroatoms on the surface of CDs improves binding affinity due to coordination chemistry principles, leading to selective and sensitive quenching responses [52]. Due to their nanoscale size and high surface area, they have more accessible active sites for metal ions. Furthermore, the eco-friendly and facile synthesis methods preserve these surface functional groups and dopants, thereby enhancing their sensing performance. Fundamentally, the high sensitivity and selectivity of these CDs arise from the combination of surface functional groups capable of selective coordination, intrinsic doping that adjusts their electronic properties, and optimized fluorescence characteristics that respond strongly to Hg^2+^ and Fe^3+^ binding. These factors together yield CDs compatible for rapid and sensitive detection of these toxic metal ions in aqueous environments.

### 3.4. Sensitivity of CDs Toward Hg^2+^

**Figure 9:**
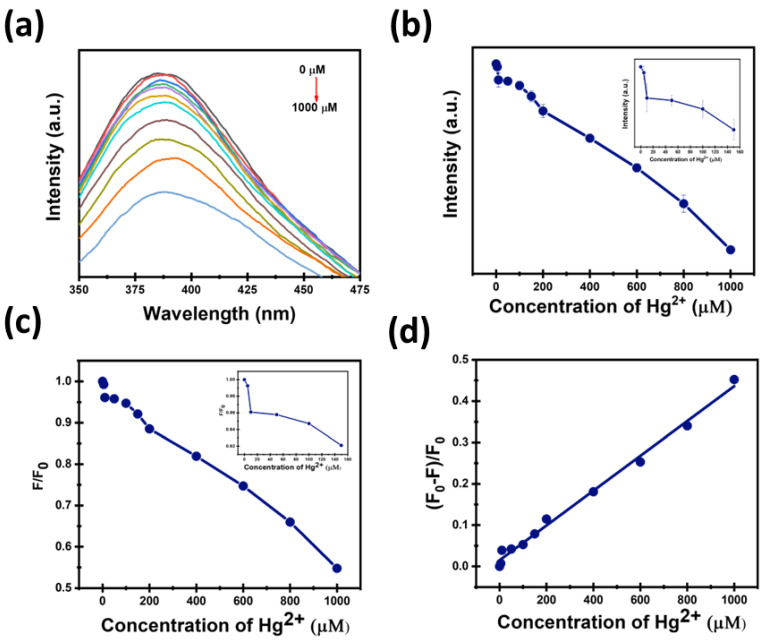
(**a**,**b**) Effect of different concentrations of Hg^2+^ on the intensity of CDs; (**c**) The ratio of fluorescence intensity to Hg^2+^ concentration (μM); (**d**) Quenching efficiency.

As shown in Figure 9a,b, the intensity of CDs decreases by adding Hg^2+^ with increasing concentrations. In Figure 9c, the addition of Hg^2+^ ions to the CDs solution leads to a clear concentration-dependent decrease in F/F_0_ ratio, indicative of fluorescence quenching. The quenching efficiency was quantitatively analyzed to investigate how the CDs fluorescence is quenched by the addition of Hg^2+^ ions, using the plot of (F_0_ − F)/F_0_ versus Hg^2+^ concentration shown in Figure 9d. This plot exhibited an excellent linear relationship within the concentration range with R^2^ = 0.99. The linearity indicates a direct proportional Hg^2+^ ions quenching effect on the CDs intensity, which enables a reliable quantification and suggests a well-defined interaction mechanism between Hg^2+^ ions and the CDs surface.

**Figure 10:**
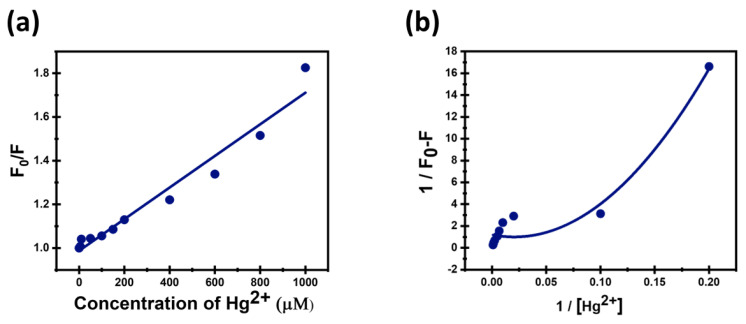
(**a**) Stern–Volmer quenching plot and (**b**) 1:2 Benesi–Hildbrand plot for CDs with Hg^2+^.

The linear increase in the Stern–Volmer plot of (F_0_ − F) versus concentration of Hg^2+^ (Figure 10a) indicates a concentration-dependent quenching of CDs’ fluorescence that follows the classical Stern–Volmer relationship:

$$\frac{F_{0}}{F}=1+K_{\mathrm{sv}}[{\mathrm{Hg}}^{2+}]$$

K_sv_ was obtained from the slope of the S-V plot and found to be equal to 7.23 M^−1^. The linear S-V plot with R^2^ = 0.96 suggests that the quenching mechanism predominantly involves dynamic quenching, where the quencher molecules collide with the excited fluorophores, causing a non-radiative deactivation proportional to quencher concentration. The LOD was estimated to be 0.009 μM, and the LOC was equal to 0.029 μM.

The determined LOD value of the present sensor demonstrates competitive sensitivity toward Hg^2+^ ions compared to other recent fluorescent sensors reported in the literature (Table 2). While several advanced sensors achieve slightly lower detection limits around 0.27–6.00 nM, the 9.58 nM LOD is notably lower than many others exhibiting detection limits in nanomolar to micromolar ranges. Therefore, the sensor exhibits reliable low-nanomolar sensitivity, suitable for environmental monitoring and practical applications.

The Benesi–Hildbrand plot displayed in Figure 10b, and the binding constant was calculated for 1:2 stoichiometry. The Benesi–Hildebrand plot shows nonlinear behavior with 1:2 stoichiometry, and a high correlation coefficient (R^2^ = 0.96) indicates that the fluorescence quenching proceeds from the formation of a complex containing one fluorophore bound to two quencher molecules. This suggests a more complex binding interaction than the simple 1:1, which can reflect stronger and multiple binding sites. Good fitting supports the assumption of this higher-order complexation contributing to the observed fluorescence quenching [59].

### 3.5. Sensitivity of CDs Toward Fe^3+^

**Figure 11:**
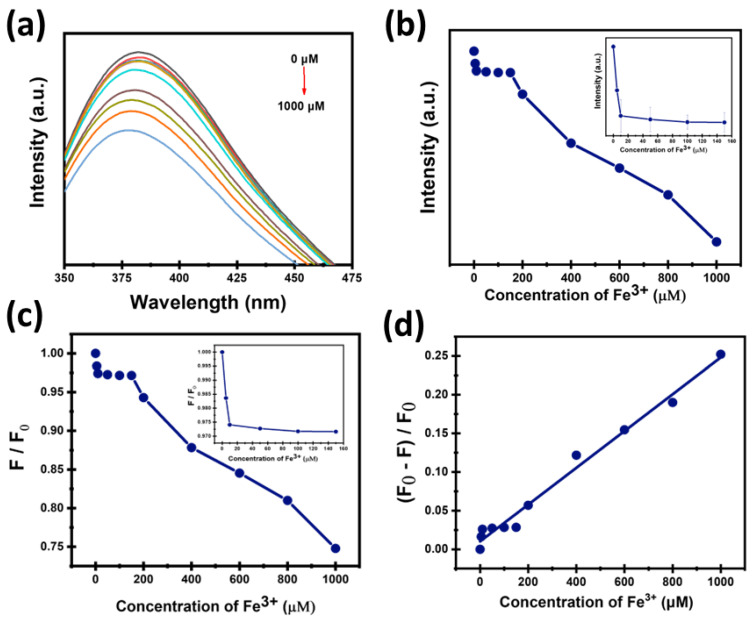
(**a**,**b**) The relationship between the CDs’ fluorescence intensity and Fe^3+^ concentration; (**c**) The ratio of fluorescence intensity to concentration (μM); (**d**) Linear relationship between the quenching efficiency of CDs and Fe^3+^ concentration.

Figure 11a,b show that the intensity of CDs decreases gradually with the increase of Fe^3+^ concentration. In Figure 11c, the fluorescence quenching efficiency of CDs by the addition of Fe^3+^ ions was quantitatively estimated by plotting the F/F_0_ versus Fe^3+^ concentration. It was noted that the F/F_0_ value decreased with the increase of Fe^3+^ concentration, indicating efficient quenching by Fe^3+^ ions. Quenching efficiency was studied to analyze how the fluorescence of CDs is quenched with Fe^3+^ ions by plotting (F_0_ − F)/F_0_ versus Fe^3+^ concentration shown in Figure 11d. This plot demonstrates a strong linear relationship with R^2^ = 0.98. The strong linearity indicates a directly proportional quenching effect of Fe^3+^ ions on the intensity of CDs, allowing reliable quantification, and suggests a well-defined interaction mechanism between Fe^3+^ ions and the surface of CDs.

**Figure 12:**
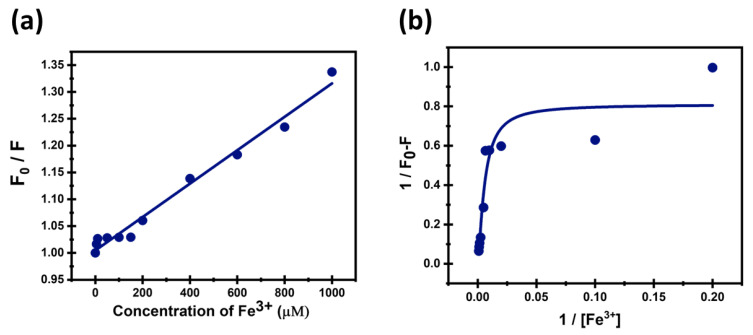
(**a**) Stern–Volmer plot and (**b**) Benesi–Hildbrand plot of CDs with Fe^3+^ ions.

The linear increase in the S-V plot (Figure 12a) indicates a concentration-dependent quenching of CDs’ fluorescence that follows the classical S-V relationship and has a K_SV_ value equal to 3.11 M^−1^. This strong linearity is characteristic of a well-defined quenching process and supports the suitability for the detection of Fe^3+^ ions by the synthesized CDs. The LOD and LOC were calculated to be 0.02 μM and 0.07 μM, respectively. Moreover, LOD of the prepared CDs sensors were compared with literature in Table 3.

The LOD of the diphenylamine-derived CDs for Fe^3+^ ion detection was calculated to be 22.27 nM. The table above illustrates their high sensitivity compared to many previously reported sensors. These values are significantly lower than the micromolar LODs exhibited by several conventional CDs (Such as 1.90 μM and 3.80 μM) and are superior to many other CDs, which display LODs ranging from 162.00 nM down to 13.80 nM. The relatively wide linear detection ranges up to 1000.00 μM further demonstrate their practical applicability for environmental and analytical Fe^3+^ monitoring. Compared with other sensors, the presented sensors show a noticeable improvement in sensitivity. This enhanced performance can be attributed to the tailored surface chemistry that facilitates the efficient fluorescence quenching by Fe^3+^ ions.

Figure 12b shows a B-H plot that has a 1:2 stoichiometry, nonlinear behavior, and a relatively good correlation coefficient (R^2^ = 0.89), proving that the quenching emerges from the complex formation, which contains one fluorophore bound to two quencher molecules. This suggests a more complex binding interaction, which might reflect stronger and more multiple binding sites. Further studies should be conducted to gain a deeper understanding of fluorescence quenching mechanisms and to study the binding interactions and pathways involved at the molecular level. Detailed mechanistic insights will help to optimize sensor design and sensitivity. Moreover, investigating potential interferences and sensor behaviors will provide robustness for real-world applications.

### 3.6. Simultaneous Detection of Hg^2+^ and Fe^3+^

**Figure 13:**
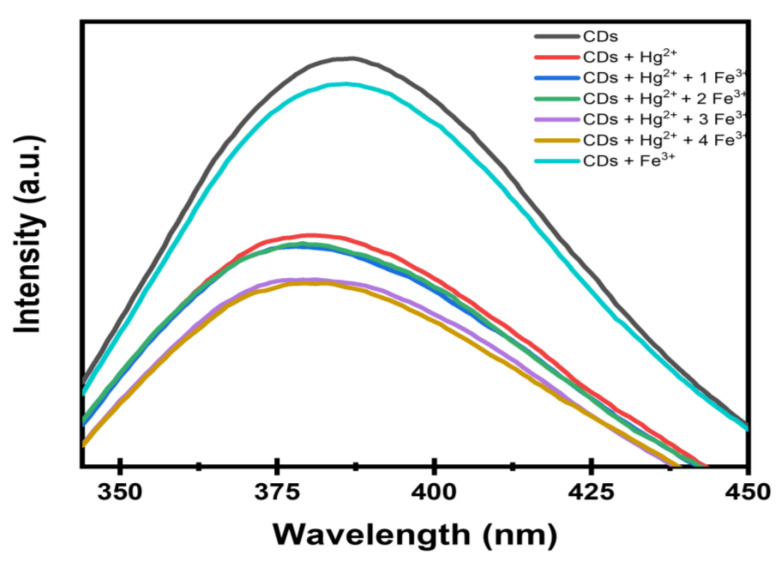
Effect of Hg^2+^ detection in the presence of Fe^3+^ ions.

The interference plot shows that Hg^2+^ produces a strong and distinct quenching of the intensity of CDs, indicating high sensitivity. As shown in Figure 13, Fe^3+^ causes only a gradual additional decrease; however, at higher concentrations, it partially masks the Hg^2+^ response. Therefore, in real samples containing large excess Fe^3+^, the Hg^2+^-induced quenching may not be distinguishable. This represents a practical limitation, and samples with high Fe^3+^ may require pretreatment or correction. In summary, Fe^3+^ ions demonstrated significant interference in the fluorescence detection of Hg^2+^ by the synthesized CDs, highlighting the need for a masking agent in practical sensing applications.

## 4. Conclusions

This study provides a rapid and facile microwave-assisted synthesis of CDs for efficient detection of Hg^2+^ and Fe^3+^ in aqueous solutions using o-diphenylamine as a carbon precursor. The prepared CDs show a high QY of 44.69%. These CDs provide sufficient sensitivity toward Hg^2+^ and Fe^3+^ ions with low LOD up to 9.58 nM and 22.27 nM, respectively. The excellent photostability, strong water solubility, and long storage time make these CDs useful in multiple applications, including bioimaging and sensing. Both Hg^2+^ and Fe^3+^ exhibit dynamic (collisional) quenching as the primary fluorescence quenching mechanism, evidenced by linear Stern–Volmer plots. Nonlinear Benesi–Hildebrand plots indicate that static quenching via ground-state complex formation does not adequately describe their interaction, suggesting alternative binding mechanisms.

## Figures and Tables

**Table 1:** Comparison of QY of CDs produced from different precursors in previous studies and this work.

Precursor	Method	QY (%)	Reference
citric acid and amino acids	Hydrothermal synthesis	36.45	[43]
citric acid and tris (hydroxymethyl)-aminomethane	Hydrothermal synthesis	37.00	[44]
1,4-phenylene diisocyanate	Solvothermal	38.00	[45]
di-2-pyridiylketone	Hydrothermal synthesis	5.55	[46]
o-phenylenediamine	Solvothermal treatment	26.70	[47]
cytidine diphosphate choline (CDPC) and ethylenediamine	Pyrolysis	25.00	[48]
nitazoxanide and 3-mercaptopropionic acid	Hydrothermal synthesis	32.00	[49]
citric acid (CA) and glutathione	Microwave-assisted	10.90	[50]
o-diphenylamine	Microwave-assisted	44.69	This work

**Table 2:** Different sensors based on CDs for Hg^2+^ detection.

Materials	Readout Mechanism	Linear Range	LOD	Reference
N-CDs	Turn-Off	0–40 μM	3.10 nM	[53]
N-CDs	Turn-Off	0.01–100 μM	6.27 nM	[54]
CD-wrapped AuNP	Turn-Off	9.0 × 10^−7^–9.0 × 10^−5^ M	281.00 nM	[55]
NS-CDs	Turn-On	0–50 × 10^−6^ M	6.77 × 10^−7^ M	[49]
HCYS	Turn-Off	-	1.41 μM.	[56]
CDs	Turn-Off	-	0.16 μM	[50]
N, S-GQDs	Turn-Off	1–30 nM 100–1000 nM	0.27 nM 36.85 nM	[57]
Cysteine-functionalized GQDs	Turn-Off	0–10 μM	20.00 nM	[58]
CDs	Turn-Off	0–1000 μM	0.00958 μM (9.58 nM)	This work

**Table 3:** Different sensors based on CDs for Fe^3+^ detection.

Materials	Readout Mechanism	Linear Range	LOD	Reference
CDs	Turn-Off	8–80 μM	3.80 μM	[60]
N-CDs	Turn-Off	0.002–8 μM	13.80 nM	[61]
CDs	Turn-Off	0–1000 μM	1.90 μM	[62]
FPCDs	Turn-Off	-	162.00 nM	[63]
N-CDs	Turn-Off	20–80 μM	3.18 μM	[64]
CDs	Turn-Off	0.2–200 μM	62.00 nM	[65]
N-CDs/OPD	Turn-Off	20–80 μM	7.12 μM	[66]
B@HRCDs	Turn-Off	0–80 μM	1.08 μM	[67]
CDs (Diphenyleneamine)	Turn-Off	0–1000 μM	0.02 μM (22.27 nM)	This work

## Data Availability

The data presented in this study are available on request from the corresponding author.
